# Early Cryoablation After First Diagnosis of Atrial Fibrillation Reduces Arrhythmia Recurrence in Heart Failure Patients

**DOI:** 10.1016/j.jacasi.2024.08.005

**Published:** 2024-09-24

**Authors:** Monami Ando, Satoshi Yanagisawa, Hirohiko Suzuki, Yukihiko Yoshida, Yasunori Kanzaki, Itsuro Morishima, Shinji Ishikawa, Yosuke Kamikubo, Satoshi Okumura, Hiroyuki Kato, Yoshiaki Mizutani, Yosuke Murase, Kosuke Nakasuka, Shunichiro Warita, Satoru Sekimoto, Yoshio Takemoto, Nobuhiro Takasugi, Shiou Ohguchi, Michiharu Senga, Kenichiro Yokoi, Ryo Watanabe, Yasuhiro Ogura, Rei Shibata, Yasuya Inden, Toyoaki Murohara

**Affiliations:** a Department of Cardiology, Japanese Red Cross Aichi Medical Center Nagoya Daini Hospital, Nagoya, Japan; b Department of Advanced Cardiovascular Therapeutics, Nagoya University Graduate School of Medicine, Nagoya, Japan; c Department of Cardiology, Nagoya University Graduate School of Medicine, Nagoya, Japan; d Department of Cardiology, Ogaki Municipal Hospital, Ogaki, Japan; e Department of Cardiology, Cardiovascular Center, Anjo Kosei Hospital, Anjo, Japan; f Department of Cardiology, Toyota Memorial Hospital, Toyota, Japan; g Department of Cardiology, Konan Kosei Hospital, Konan, Japan; h Division of Cardiology, Japan Community Healthcare Organization Chukyo Hospital, Nagoya, Japan; i Department of Cardiology, Yokkaichi Municipal Hospital, Yokkaichi, Japan; j Department of Cardiology, Komaki City Hospital, Komaki, Japan; k Department of Cardiology, Nagoya City University Graduate School of Medical Sciences, Nagoya, Japan; l Department of Cardiology, Gifu Prefectural General Medical Center, Gifu, Japan; m Department of Cardiology, Nagoya City University East Medical Center, Nagoya, Japan; n Department of Cardiology, Gifu Prefectural Tajimi Hospital, Tajimi, Japan; o Division of Cardiovascular Medicine, Gifu University Hospital, Gifu, Japan; p Department of Cardiology, Kasugai Municipal Hospital, Kasugai, Japan; q Department of Cardiology, Kuwana City Medical Center, Kuwana, Japan; r Department of Cardiology, Kainan Hospital, Yatomi, Japan

**Keywords:** atrial fibrillation, catheter ablation, cryoballoon ablation, early ablation heart failure

## Abstract

**Background:**

Atrial fibrillation (AF) and heart failure (HF) often coexist, leading to increased mortality. A cryoballoon-based approach is a potential treatment for patients with HF because of its safety and efficacy.

**Objectives:**

The authors sought to evaluate the optimal timing of cryoballoon ablation after the first clinical diagnosis of AF and its prognosis for patients with HF.

**Methods:**

This large-scale multicenter study retrospectively collected data of patients with HF who underwent cryoballoon ablation for AF from 17 Japanese institutions. Patients were divided into 2 groups depending on the duration between the first diagnosis and ablation using a median time of 0.5 year (IQR: 0.3-2.0 years). Clinical endpoints of recurrence, mortality, and HF hospitalization were compared between the 2 groups.

**Results:**

Among 3,655 patients, 543 with HF were included for analysis. During a median follow-up period of 21.3 months (IQR: 12.0-36.8 months), 151 of 520 patients (29%) had a recurrence. The AF recurrence rate was significantly lower in the early-ablation group (≤0.5 year) than in the delayed-ablation group (>0.5 year) (24% [65/266] vs 34% [86/254], respectively; *P* = 0.018). In the multivariable analysis, early ablation ≤ 0.5 year was independently associated with an absence of recurrence (HR: 0.581; 95% CI: 0.401-0.842; *P* = 0.004). Delayed time for cryoballoon ablation incrementally increased the risk of postablation recurrence. Antiarrhythmic drug use was independently associated with delayed ablation. No significant differences in mortality or HF hospitalization were observed between the 2 groups.

**Conclusions:**

Early cryoablation reduced the risk of recurrence in patients with HF, which may help improve clinical management.

Atrial fibrillation (AF) is the most frequent arrhythmia and is associated with significant morbidity and mortality.^1^ Additionally, heart failure (HF) is often accompanied with AF. These conditions increase the risk of hospitalization, morbidity, and mortality as well as the deterioration of health and quality of life.^2^

The treatment of AF is currently shifting to early intervention after its diagnosis. The EAST-AFNET 4 (Early Rhythm Control Therapy in Patients with Atrial Fibrillation) trial demonstrated that early rhythm control therapy reduces the risk of adverse cardiovascular outcomes more than conventional therapy among patients with early AF and cardiovascular conditions.^3^ As for the catheter ablation approach, increased time between the first AF diagnosis and catheter ablation may adversely affect long-term clinical efficacy, and early intervention by catheter ablation increases success rates.^4^ However, this may not be applicable to patients with HF, because catheter ablation for these patients may cause complications perioperatively. Moreover, HF causes deterioration of cardiac functions and requires administration of cardiac protection drugs, resulting in a difficulty in timing its relevant intervention.^5^

Cryoballoon ablation can achieve pulmonary vein (PV) isolation with a high procedural success rate, short procedural duration, and durability and may be more feasible and applicable than other therapies for patients with HF.^6^ Although we previously demonstrated that cryoballoon ablation for AF in HF patients was feasible and effective in significantly improving cardiac function in a large-scale multicenter study,^7^ to the best of our knowledge, few studies have investigated the optimal timing of cryoballoon ablation for patients with HF.^8^

The present study investigated the impact of the duration between the first AF diagnosis and cryoballoon ablation on the outcomes of HF patients. We also evaluated the underlying causes for variation in the timing of ablation between patients.

## Methods

### Study population

The study population was retrospectively recruited from a database of patients with HF who underwent cryoballoon ablation across 17 institutions in Japan. The protocol for the recruitment of the study population was described previously.^7^ Briefly, all consecutive patients with HF who underwent cryoballoon ablation for AF in 2014 to 2019 at the participating hospitals were included in the analysis. Patients with unknown duration between the AF diagnosis and ablation were excluded. The indications for catheter ablation for AF complied with recent guidelines.^9^ Cryoballoon ablation was selected at the operators’ discretion at each institution according to patient characteristics and anatomy of their left atrium (LA) and PV.

Informed consent for the procedure was obtained from all patients. The study protocol was approved by each institution’s institutional ethics committee, and the study was performed in accordance with the principles of the Declaration of Helsinki.

### Definition of HF and duration of AF

In general, HF was defined as a previous history of hospitalization or diagnosis of HF, reduced left ventricular ejection fraction (LVEF) ≤40% at baseline, continuous administration of diuretics to control congestive HF, and elevated B-type natriuretic peptide (BNP) levels with any related symptoms of HF. Patients with only high BNP levels were not included in the study population because of the absence of further supportive evidence of the presence of HF such as the relevant symptoms and decreased cardiac function. The final inclusion of the HF patients was decided by each institution’s chief investigator after reviewing the patients’ medical records. Duration of AF was defined as the time from first diagnosis of AF detected at any examination (electrocardiogram, Holter monitoring, or device interrogation) to the date of the catheter ablation procedure.

### Catheter ablation procedure

Before the cryoballoon procedure, transesophageal echocardiography and/or contrast-enhanced computed tomography were performed to exclude the presence of LA thrombus and to clarify the anatomy of the LA and PV, if applicable. Oral anticoagulant drugs were administered >3 weeks before the ablation. Periprocedural management of anticoagulation and antiarrhythmic drug (AAD) prescriptions were determined by each institution.

The ablation procedure was performed with the patient under minimal to moderate sedation. An activated clotting time was maintained between 300 and 350 seconds by heparin infusion adjustment during the procedure. After trans-septal puncture, a 28-mm second-generation cryoballoon catheter (Arctic Front Advance, Medtronic Inc) was positioned at the PV ostium using a 12-F steerable sheath (Flex Cath Advance, Medtronic Inc). A duty cycle cryoablation of 120 to 240 seconds in each PV was repeatedly applied while monitoring PV potentials with a spiral-mapping catheter (Achieve, Medtronic Inc). A high-output stimulation of the right phrenic nerve was applied during the right PV cryoablation. When the PV potential remained after repetitive cryoballoon ablations, a touch-up ablation was added using a radiofrequency or cryoablation catheter. An additional ablation of the liner ablations and substrate ablation were performed according to the discretion of the attending operators, if necessary.

### Follow-up and assessment

Patients were followed up at the outpatient clinic of each institution. At each follow-up visit, patients underwent surface 12-lead electrocardiography or Holter monitoring examinations to check for recurrence. Additional long-term Holter monitoring was scheduled when patients reported palpitations with suspected recurrence but showed no evidence of recurrence during routine follow-up examinations, if necessary. Device interrogation data and insertable cardiac monitoring were also used to detect recurrence. Recurrence monitoring was not standardized and was left to the discretion of the institutions. The typical follow-up examination schedules to assess recurrence in all institutions are listed in Supplemental Table 1. AF recurrence was defined as any atrial tachyarrhythmias lasting >30 seconds detected after the 3-month blanking period, regardless of AAD administration. The study assessed occurrences of all-cause death and HF hospitalization (first admission) after the ablation performance in all patients. The latest follow-up data of echocardiography and BNP levels were also obtained. Patient characteristics, procedural data, and outcomes were collected from the patients’ medical records from each institution.

The outcome of the study was the difference in prognosis (recurrence, HF hospitalization, and death) after the ablation according to the duration between AF diagnosis and catheter ablation. We also assessed changes in echocardiographic parameters and BNP levels among the groups.

### Statistical analysis

Continuous data are presented as mean ± SD or median (IQR). Categorical values are expressed as numbers (percentages). Comparisons of the differences in the baseline characteristics were analyzed using a Student’s *t*-test for parametric data and the Mann-Whitney *U* test for nonparametric data. Categorical variables were compared using the chi-squared test or Fisher exact test. Differences between the baseline and follow-up outcome parameters were compared using a paired *t*-test, whereas the nonparametric Wilcoxon signed-rank test was used to perform paired comparisons of the parameters. Kaplan-Meier survival curves for event-free rates were estimated and compared using a log-rank test. Mixed-effects Cox proportional hazards analysis was conducted to evaluate predictors of recurrence. Institution was treated as a random factor and the proportion of participants was modeled using a multilevel approach in both univariable and multivariable analyses to account for institution-dependent variables. Variables included in the multivariable analysis were selected based on their presumed relationship to the outcome and potential confounding effects. Multivariable analyses were performed using the forced-entry method for these variables. Multicollinearity was assessed using the variance expansion factor, with a variance expansion factor ≥5.0 indicating problematic multicollinearity. The proportional hazards assumption was tested using the -log (log [survival]) plot for each variable. Additionally, mixed-effects logistic regression analysis was performed as for the Cox proportional hazards mixed model described above to evaluate factors associated with the late-treatment group. An adjusted Cox proportional hazards model was used to estimate the average relative treatment effect HRs with associated 95% CIs. The effect of continuous values on the early-ablation vs late-ablation HR was estimated by including an interaction term in the models. Statistical significance was set at *P* < 0.05. All analyses were conducted using SPSS version 29.0 (SPSS Inc) and R (version 4.3.3).

## Results

### Baseline patient characteristics and procedural results

Among 3,655 patients from 17 institutions who underwent cryoballoon ablation for AF during the study period, 549 (15%) were preoperatively diagnosed with HF. Of them, 6 patients were excluded because of an unknown time from diagnosis of AF, resulting in 543 patients included in the analysis. Mean patient age was 69.2 ± 10.0 years, and 57% of the patients were men. Furthermore, 72% of the population had paroxysmal AF. The mean LVEF was 56.2 ± 12.8%, and 153 patients (28%) had a reduced LVEF (<50%). The median AF duration (time from AF diagnosis to ablation) in the total population was 0.5 year (IQR: 0.3-2.0 years). The population was divided into 2 groups according to the median AF duration: patients undergoing an ablation in ≤0.5 year (early-ablation group, n = 278) and patients undergoing an ablation in >0.5 year (delayed-ablation group, n = 265). Comparisons of the baseline characteristics between the 2 groups are shown in Table 1. The early-ablation group had a lower prevalence of AAD use than the delayed-ablation group (29% [80/278] vs 45% [118/265]; *P* < 0.001). The NYHA functional class (1.3 ± 0.5 vs 1.4 ± 0.6; *P* = 0.017) and CHADS_2_ score (2.2 ± 1.1 vs 2.4 ± 1.1; *P* = 0.041) were significantly lower in the early group than in the delayed group. In contrast, the LVEF was significantly higher in the early group than in the delayed group (57.6% ± 12.0% vs 54.8% ± 13.4%; *P* = 0.009). There was a significant difference in HF etiology between the groups.

**Table 1:** Comparison of Baseline Characteristics of Patients With HF

		AF Ablation		
	HF Patients With AF (n = 543)	≤0.5 Year (n = 278)	>0.5 Year (n = 265)	*P* Value
Age, y	69.2 ± 10.0	70.0 ± 8.8	68.5 ± 11.1	0.074
Male	311 (57)	152 (55%)	159 (60)	0.210
Body weight, kg	62.1 ± 12.5	61.3 ± 12.8	63.0 ± 12.1	0.140
Body mass index, kg/m^2^	23.7 ± 3.9	23.6 ± 4.1	23.9 ± 3.7	0.379
Duration of AF (time from first occurrence to ablation), y	0.5 (0.3-2.0)	0.3 (0.2-0.4)	2.0 (1.0-4.0)	<0.001
AF type				<0.001
Paroxysmal	390 (72)	203 (73)	187 (71)	
Persistent	129 (24)	75 (27)	54 (20)	
Longstanding persistent	24 (4.4)	0 (0)	24 (9.1)	
Antiarrhythmic drugs	198 (37)	80 (29)	118 (45)	<0.001
Class I	109 (20)	48 (17)	61 (23)	
Class III	89 (16)	32 (12)	57 (22)	
NYHA functional class	1.4 ± 0.6	1.3 ± 0.5	1.4 ± 0.6	0.017
Comorbidity				
Hypertension	338 (62)	170 (61)	168 (63)	0.590
Diabetes mellitus	118 (22)	61 (22)	57 (22)	0.903
Coronary artery disease	61 (11)	34 (12)	27 (10)	0.451
Stroke/transient ischemic attack	61 (11)	30 (11)	31 (12)	0.738
Hemodialysis	18 (3.3)	9 (3.2)	9 (3.4)	0.918
Echocardiographic data				
Left atrial diameter, mm	40.5 ± 6.1	40.2 ± 6.3	40.8 ± 6.0	0.234
Left ventricular end-diastolic diameter, mm	48.4 ± 8.8	47.9 ± 7.5	48.9 ± 9.9	0.173
Left ventricular end-systolic diameter, mm	33.7 ± 8.1	33.1 ± 7.5	34.3 ± 8.7	0.078
Left ventricular ejection fraction, %	56.2 ± 12.8	57.6 ± 12.0	54.8 ± 13.4	0.009
≤40%	71 (13)	28 (10)	43 (16)	
40%-49%	82 (15)	42 (15)	40 (15)	
≥50%	390 (72)	208 (75)	182 (69)	
CHADS_2_ score	2.3 ± 1.1	2.2 ± 1.1	2.4 ± 1.1	0.041
CHA_2_DS_2_-VASc score	3.5 ± 1.5	3.5 ± 1.5	3.5 ± 1.5	0.881
Laboratory data				
Creatinine clearance, mL/min	63.3 ± 28.6	62.3 ± 27.8	66.1 ± 30.0	0.499
B-type natriuretic peptide levels, pg/dL	154 (104-263)	154 (103-254)	155 (106-271)	0.831
Direct-acting oral anticoagulants	477 (88)	254 (92)	233 (84)	0.007
History of device implantation				0.107
Pacemaker	24 (4.4)	9 (3.2)	15 (5.7)	
Implantable cardioverter-defibrillator	12 (2.2)	5 (1.8)	7 (2.6)	
Cardiac resynchronization therapy	4 (0.7)	4 (1.4)	0 (0)	
Medications				
Angiotensin-converting enzyme inhibitors/angiotensin receptor blockers	262 (48)	138 (50)	124 (47)	0.507
Beta-blocker	368 (68)	187 (67)	181 (68)	0.796
Spironolactone	103 (19)	51 (18)	52 (20)	0.704
Diuretic	206 (38)	104 (37)	102 (39)	0.795
Etiology of HF				<0.001
Tachycardia-induced (AF-related) cardiomyopathy	364 (67)	156 (56)	208 (79)	
Ischemic cardiomyopathy	95 (18)	79 (28)	16 (17)	
Dilated cardiomyopathy	15 (2.8)	9 (3.2)	6 (2.3)	
Hypertrophic cardiomyopathy	18 (3.3)	10 (3.6)	8 (3.0)	
Valvular heart disease	25 (4.6)	13 (4.7)	12 (4.5)	
Sarcoidosis	0 (0)	0 (0)	0 (0)	
Amyloidosis	2 (0.4)	1 (0.4)	1 (0.4)	
Other	24 (4.4)	10 (3.6)	14 (5.3)	

Values are mean ± SD, n (%), or median (IQR).

AF = atrial fibrillation; HF = heart failure.

A comparison of the procedural details between the 2 groups is demonstrated in Table 2. Notably, PV isolation was completed in all patients. Few patients had additional LA ablations in the early and delayed groups (4.7% [13/278] vs 7.9% [21/265]; *P* = 0.118). No significant difference in major complications was observed between the 2 groups (4.3% [12/278] vs 3.8% [10/265]; *P* = 0.748). All phrenic nerve injuries recovered during the follow-up period without serious symptoms.

**Table 2:** Procedural Details of Patients With HF

	AF Ablation		
	≤0.5 Year (n = 278)	>0.5 Year (n = 265)	*P* Value
Successful pulmonary vein isolation	278 (100)	265 (100)	N/A
Touch-up for incomplete pulmonary vein isolation of cryoballoon	85 (31)	69 (26)	0.241
Additional ablations			
Linear ablations in left atrium (roof, bottom, and mitral isthmus lines)	13 (4.7)	21 (7.9)	0.118
Superior vena cava isolation	3 (1.1)	2 (0.8)	0.999
Cavotricuspid isthmus ablation	202 (73)	204 (77)	0.247
Complex fractionated atrial electrogram ablation	0 (0)	0 (0)	N/A
Other ablations for another arrhythmias	3 (1.1)	4 (1.5)	0.719
Session time, min (from puncture to session end)	135.2 ± 42.0	133.4 ± 39.3	0.634
Major complications			
Overall	12 (4.3)	10 (3.8)	0.748
Stroke	1 (0.4)	0 (0)	
Transient ischemic attack	0 (0)	0 (0)	
Cardiac tamponade	2 (0.7)	2 (0.8)	
Phrenic nerve injury (persisting >1 month)	5 (1.8)	5 (1.9)	
Pseudoaneurysm	3 (1.1)	0 (0)	
Arteriovenous fistula	0 (0)	3 (1.1)	
Pneumothorax	0 (0)	0 (0)	
Prolonged hospitalization due to worsened HF	1 (0.4)	1 (0.4)	
Groin hematoma requiring blood transfusion	1 (0.4)	0 (0)	

Values are n (%) or mean ± SD.

Abbreviations as in Table 1.

### Prognosis and clinical outcomes after the ablation

**Figure 1:**
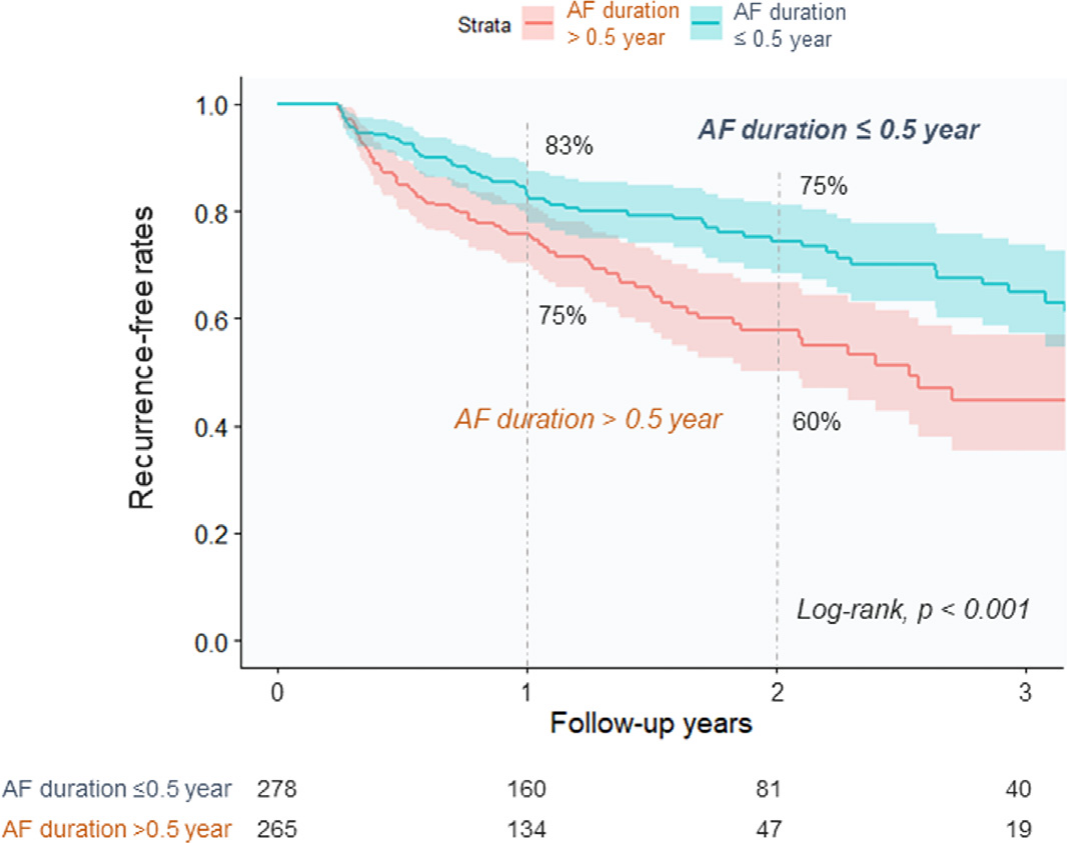
Recurrence After Cryoablation Kaplan-Meyer survival curves of recurrence after ablation between the early- (≤0.5 year) and delayed- (>0.5 year) ablation groups. AF = atrial fibrillation.

**Figure 2:**
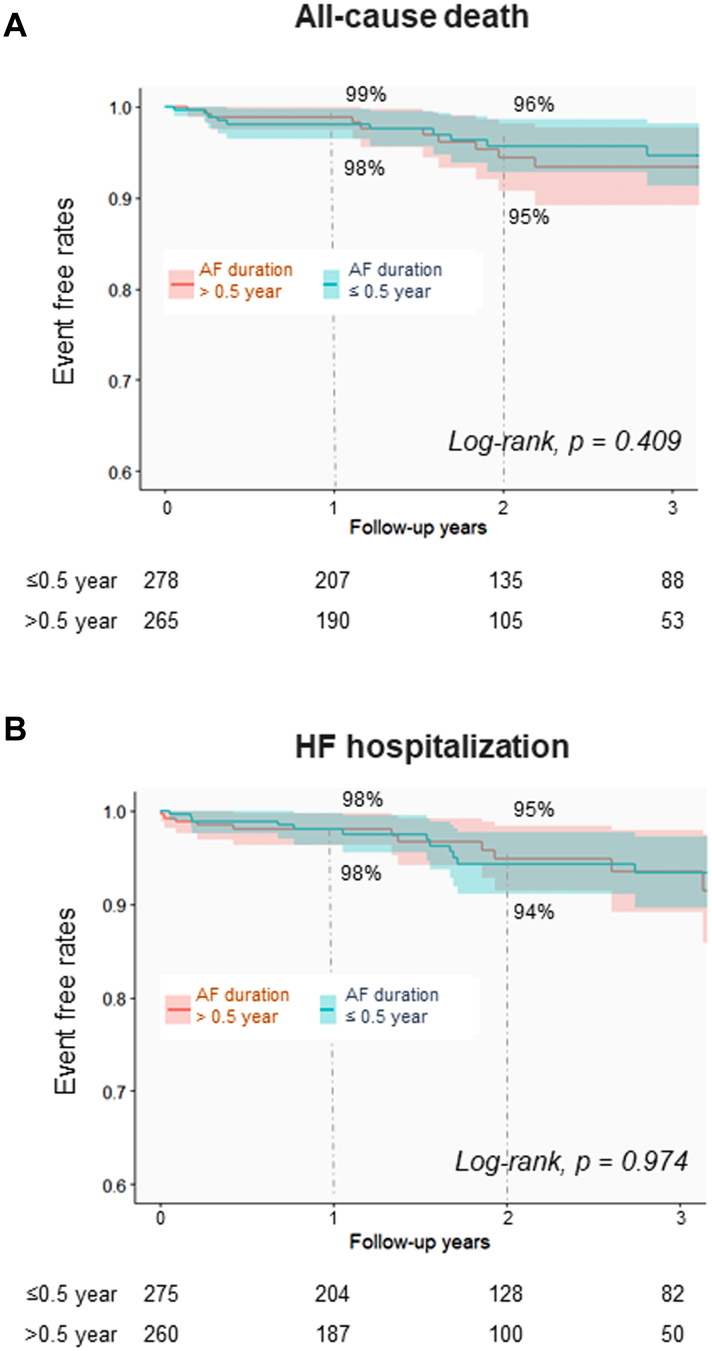
Mortality and HF Hospitalization After Cryoablation Kaplan-Meyer survival curves of all-cause death (A) and HF hospitalization (B) after ablation between the early- and delayed-ablation groups. AF = atrial fibrillation; HF = heart failure.

During a median follow-up period of 21.3 months (IQR: 12.0-36.8 months), 151 of 520 patients (29%) experienced postablation recurrence. The early group demonstrated a significantly lower recurrence rate than the delayed group (24% [65/266] vs 34% [86/254]; *P* = 0.018). Kaplan-Meier survival curves demonstrated a significant difference in recurrence-free rates after ablation between the early and delayed groups (1 year: 83% [95% CI: 79%-87%] vs 75% [95% CI: 69%-81%]; 2 years, 75% [95% CI: 69%-81%] vs 60% [95% CI: 52%-67%]), respectively (Figure 1). In contrast, there were no significant differences in all-cause death (3.6% [10/278] vs 4.5% [12/265]; *P* = 0.582) or HF hospitalization (5.1% [14/275] vs 4.6% [12/260]; *P* = 0.798) between the early and delayed groups, respectively (Table 3, Figure 2).

**Table 3:** Clinical Endpoints During the Follow-Up Period

	AF Ablation		
	≤0.5 Year (n = 278)	>0.5 Year (n = 265)	*P* Value
Early recurrence^a^	66 (24)	67 (26)	0.533
Recurrence^a^	65 (24)	86 (34)	0.018
Repeat ablation for the recurrence	47 (17)	54 (21)	0.289
Atrioventricular nodal ablation	0 (0)	0 (0)	N/A
Antiarrhythmic drug administration^a^	66 (24)	90 (34)	0.007
All-cause death	10 (3.6)	12 (4.5)	0.582
Cardiovascular death	3 (1.1)	3 (1.1)	0.999
HF hospitalization^a^	14 (5.1)	12 (4.6)	0.798

Values are n (%).

Abbreviations as in Table 1.

a Early recurrence, recurrence, antiarrhythmic drug administration, and HF hospitalization data were available for 537, 520, 535, and 535 patients, respectively.

Mixed-effects univariable Cox regression analysis demonstrated that AF duration ≤0.5 year was significantly associated with AF recurrence after the ablation. Subsequent multivariable analysis revealed that AF duration ≤0.5 year (HR: 0.581; 95% CI: 0.401-0.842; *P* = 0.004) was independently associated with recurrence in addition to body mass index, BNP levels, and LA diameter (Table 4).

**Table 4:** Predictors of Recurrence After Cryoballoon Ablation in Patients With HF

	Univariable Analysis		Multivariable Analysis		Variance Inflation Factor
	HR (95% CI)	*P* Value	HR (95% CI)	*P* Value	
Age, y	0.985 (0.968-1.002)	0.075	0.983 (0.956-1.010)	0.216	2.04
Male	0.901 (0.652-1.245)	0.528	0.887 (0.592-1.330)	0.562	1.19
Body mass index, kg/m^2^	1.003 (0.963-1.044)	0.893	0.936 (0.884-0.992)	0.025	1.65
AF duration (time from first diagnosis to ablation) ≤0.5 y	0.617 (0.440-0.865)	0.005	0.581 (0.401-0.842)	0.004	1.21
Creatinine clearance level, mL/min	1.004 (0.998-1.010)	0.201	1.008 (0.999-1.018)	0.087	2.17
B-type natriuretic peptide, pg/dL	1.001 (1.000-1.001)	0.054	1.001 (1.000-1.002)	0.042	1.31
Left atrium diameter, mm	1.026 (1.000-1.053)	0.054	1.047 (1.012-1.082)	0.008	1.44
Left ventricular ejection fraction, %	1.009 (0.994-1.025)	0.230	1.017 (0.996-1.037)	0.107	1.57
Left ventricular end-diastolic diameter, mm	0.995 (0.971-1.019)	0.666	0.987 (0.956-1.019)	0.436	1.34
CHADS_2_ score	1.014 (0.874-1.176)	0.853	N/A		N/A
CHA_₂_DS_₂_-VASc score	0.968 (0.865-1.082)	0.566	1.071 (0.914-1.256)	0.394	1.35
Persistent AF	0.980 (0.677-1.418)	0.914	0.942 (0.633-1.402)	0.768	1.12
NYHA functional class	0.837 (0.595-1.179)	0.308	0.906 (0.621-1.322)	0.609	1.21
Structural heart disease	0.958 (0.660-1.391)	0.822	0.952 (0.631-1.435)	0.813	1.38
History of device implantation	1.488 (0.931-2.378)	0.097	1.479 (0.893-2.451)	0.128	1.07
Aggressive sites (≥50% of patients with early ablation)	0.891 (0.534-1.488)	0.659	1.271 (0.693-2.333)	0.438	1.46

Mixed-effects Cox proportional hazards analysis with institution as a random effect and “aggressive sites” variable incorporated as a hierarchical model. CHADS_2_ score was excluded because it is highly collinear with CHA_2_DS_2_-VASc score in multivariable analysis model.

Abbreviations as in Table 1.

**Figure 3:**
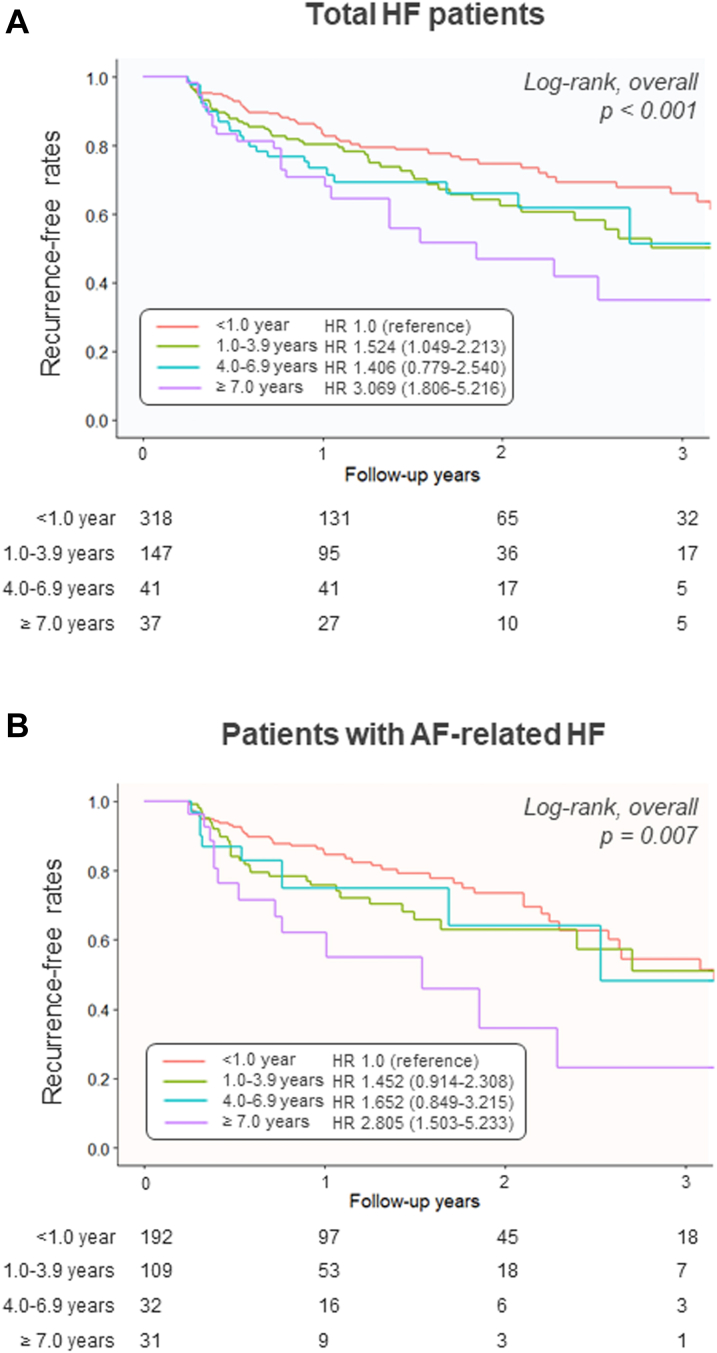
Increased Recurrence Risk Across the Time to Ablation Kaplan-Meyer survival curves of recurrence-free rates among the 4 groups according to AF duration (<1.0 year, 1.0-3.9 years, 4.0-6.9 years, and ≥7.0 years) in the (A) total ablation group and (B) patients with AF-related HF. Abbreviations as in Figure 2.

The risk of AF recurrence according to 4 categorized groups divided by AF duration (<1.0 year, 1.0-3.9 years, 4.0-6.9 years, and ≥7.0 years) is shown in Figure 3. Delayed ablation incrementally increased the risk of postablation recurrence in the total population (log-rank, *P* < 0.001 overall) (Figure 3A). This significant trend was also observed in 364 patients with AF-related HF (log-rank, *P* = 0.007 overall) (Figure 3B).

**Figure 4:**
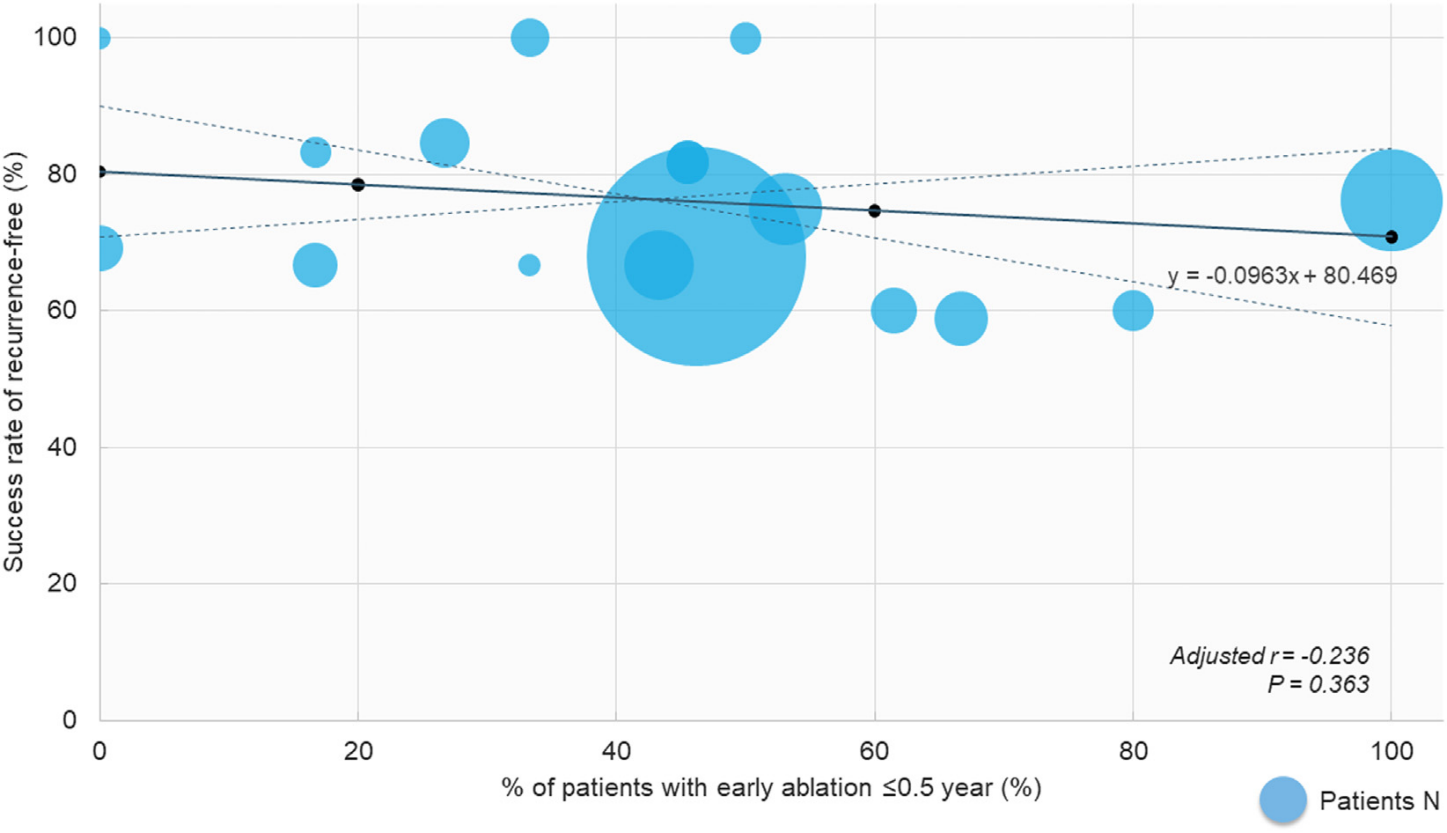
Success Rate for Cryoablation Among Institutions The success rate for recurrence-free ablation and percentage of patients with early ablation ≤0.5 year in all institutions. The trend line was calculated with an adjustment to the number of the patients in each institution with 95% CIs (dotted lines). Sample-size weighted correlation coefficients and *P* values are presented.

The proportion of patients in the early-ablation group varied among the institutions; however, the success rate for recurrence-free ablation did not differ remarkably (Figure 4). Additionally, sample size–weighted analysis revealed no significant correlation between the success rate for recurrence-free ablation and percentage of patients with early ablation ≤0.5 year in all institutions (adjusted *r* = –0.236, *P* = 0.363) (Figure 4).

In addition, we defined an aggressive institution as that enrolling a study population with ≥50% of the patients undergoing early ablation and at least ≥10 patients. A total of 128 patients at 5 institution sites were assigned to the aggressive institution group. The aggressive institution was not significantly associated with recurrence-free ablation in multivariable analysis (HR: 1.271; 95% CI: 0.693-2.333; *P* = 0.438) (Table 4).

### Changes in echocardiography and BNP levels after ablation in the groups

**Figure 5:**
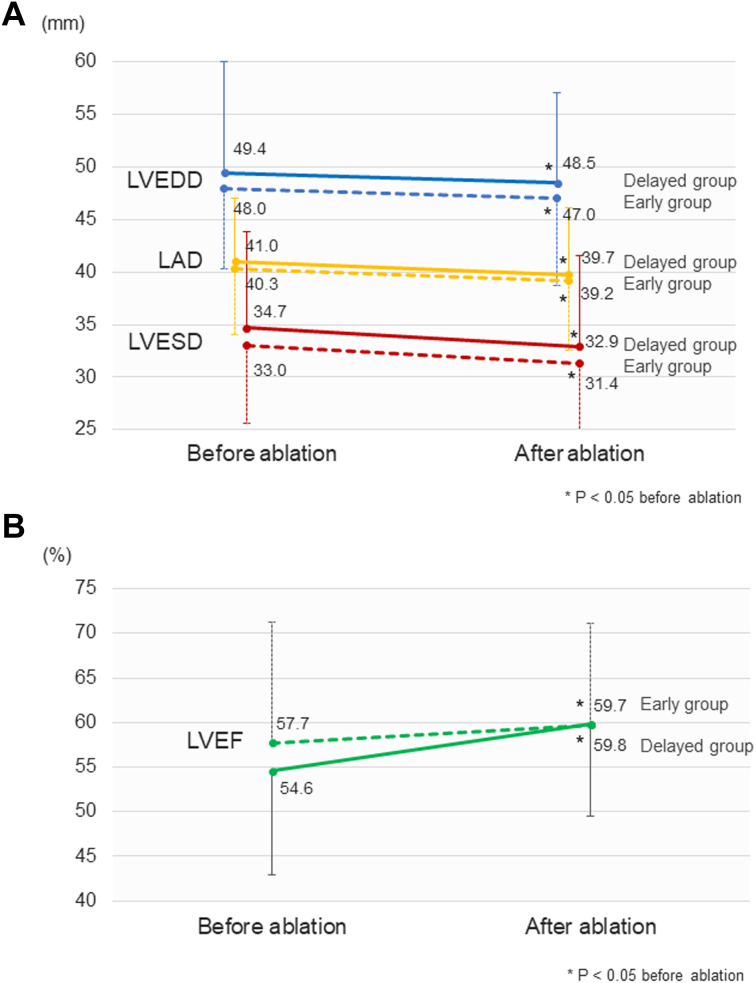
Echocardiography Changes Before and After Ablation Changes in the (A) LAD, LVEDD, and LVEDS and (B) LVEF from baseline to postablation in the early- and delayed-ablation groups. LAD = left atrial diameter; LVEDD = left ventricular end-diastolic diameter; LVEDS = left ventricular end-systolic diameter; LVEF = left ventricular ejection fraction. Values are the mean ± SD.

**Figure 6:**
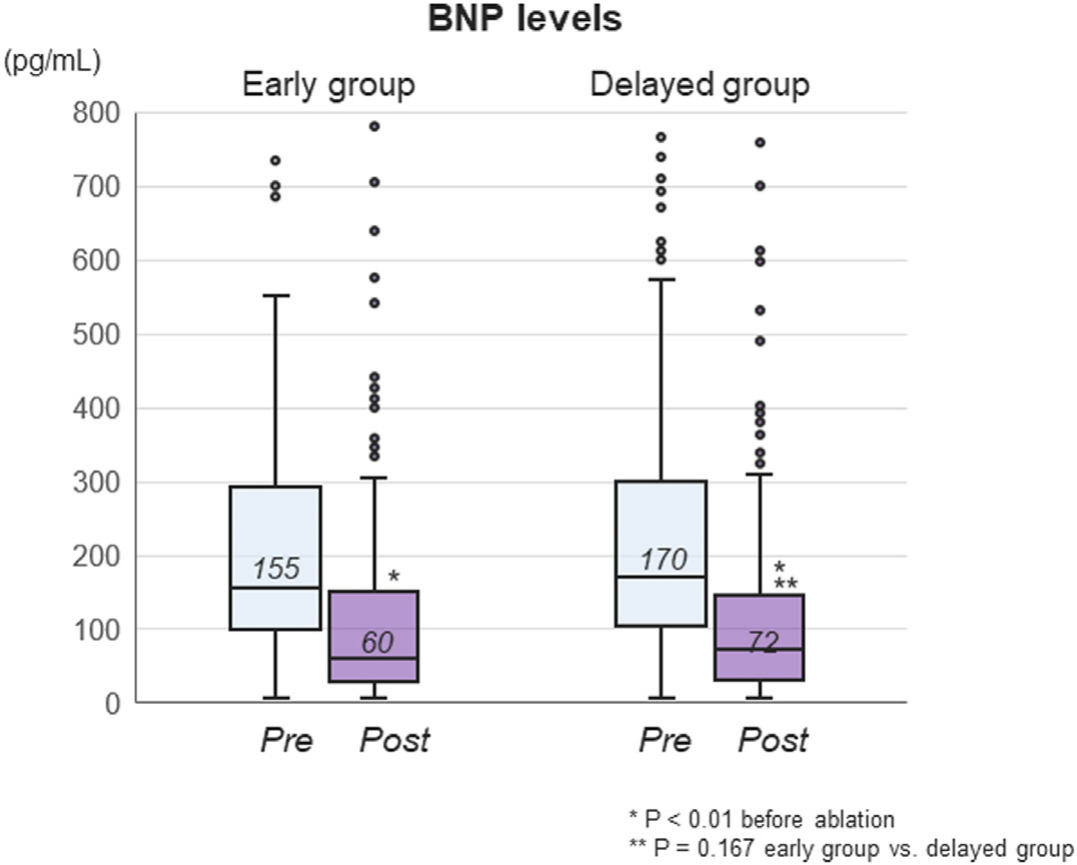
BNP Levels After Cryoablation Changes in BNP levels from baseline to follow-up postablation between the early- and delayed-ablation groups. BNP = B-type natriuretic peptide.

Echocardiography and BNP levels were obtained during the follow-up period (≥30 days after ablation) in 433 and 368 patients, respectively. Both the early and delayed groups exhibited a significant improvement of LA and left ventricular diameters from before to after the ablation with a median follow-up of 12.2 months (IQR: 5.7-26.2 months) (Figure 5A). However, absolute echocardiography values were relatively higher at any time point in the delayed group than in the early group, and these values changed in a parallel manner after the procedure without closing the gap present between them at baseline. The LVEF was significantly improved in both groups (Figure 5B). Similarly, the BNP levels significantly decreased after a median follow-up of 14.4 months (IQR: 7.3-28.1 months) in the early-ablation group (155 pg/mL [IQR: 97-292 pg/mL] to 60 pg/mL [IQR: 28-149 pg/mL]; *P* < 0.001) and in the delayed-ablation group (170 pg/mL [IQR: 102-300 pg/mL] to 72 pg/mL [IQR: 30-144 pg/mL]; *P* < 0.001) (Figure 6).

### Baseline characteristics associated with delayed ablation

Mixed-effects multivariable logistic regression analysis demonstrated that AAD use (OR: 1.648; 95% CI: 1.090-2.491; *P* = 0.018) was independently associated with delayed ablation (Table 5). Among 193 patents receiving preoperatively administered AADs, 126 were recurrence-free after the ablation. Of them, 77 and 49 patients discontinued and continued AADs after the ablation, respectively. The prognoses of mortality and HF hospitalization did not significantly differ between the discontinued and continued groups (log-rank, *P* = 0.419 and *P* = 0.939, respectively) (Supplemental Figure 1). Similar prognoses were also observed when focusing the population separately into the early- and delayed-ablation groups.

**Table 5:** Characteristics Associated With Delayed Ablation

	Univariable Analysis		Multivariable Analysis		
	OR (95% CI)	*P* Value	OR (95% CI)	*P* Value	Variance Inflation Factor
Age, y	0.979 (0.960-0.998)	0.033	0.973 (0.943-1.004)	0.089	2.40
Male	1.451 (0.996-2.115)	0.053	1.252 (0.805-1.947)	0.318	1.25
Body mass index, kg/m^2^	1.045 (0.995-1.097)	0.081	1.028 (0.964-1.095)	0.401	1.59
Creatinine clearance level, mL/min	1.005 (0.998-1.012)	0.132	1.001 (0.990-1.012)	0.823	2.17
B-type natriuretic peptide, pg/dL	1.000 (0.999-1.001)	0.896	1.000 (0.999-1.001)	0.813	1.31
Left atrium diameter, mm	1.008 (0.978-1.040)	0.591	0.985 (0.948-1.024)	0.449	1.44
Left ventricular end-diastolic diameter, mm	1.028 (1.000-1.057)	0.048	1.021 (0.985-1.058)	0.260	1.33
CHADS_2_ score	1.118 (0.941-1.329)	0.205	N/A	N/A	N/A
CHA_2_DS_2_-VASc score	0.955 (0.841-1.084)	0.475	1.135 (0.940-1.371)	0.189	1.97
Persistent AF	1.003 (0.653-1.539)	0.991	1.016 (0.633-1.630)	0.948	1.12
NYHA structural class	0.939 (0.651-1.354)	0.737	0.931 (0.607-1.428)	0.743	1.19
Structural heart disease	1.341 (0.854-2.107)	0.202	1.620 (0.977-2.686)	0.061	1.09
History of device implantation	1.352 (0.675-2.708)	0.395	1.413 (0.671-2.975)	0.363	1.08
Antiarrhythmic drug use	1.655 (1.120-2.445)	0.011	1.648 (1.090-2.491)	0.018	1.09
Left ventricular ejection fraction <50%	0.940 (0.598-1.478)	0.788	1.001 (0.574-1.746)	0.998	1.35

Mixed-effects logistic regression analysis with institution as a random effect. CHADS_2_ score was excluded because it is highly collinear with CHA_2_DS_2_-VASc score in multivariable analysis model.

Abbreviations as in Table 1.

### Subanalysis in patients with LVEF ≥50% and <50%

The total population was divided into 2 groups: 390 patients with LVEF ≥50% and 153 patients with LVEF <50%. Patients with LVEF ≥50% experienced significantly fewer HF hospitalizations than those with LVEF <50% (log-rank, *P* = 0.001), whereas no significant difference in recurrence or mortality was observed between the 2 groups (log-rank, *P* = 0.320 and *P* = 0.852, respectively) (Supplemental Figure 2).

**Figure 7:**
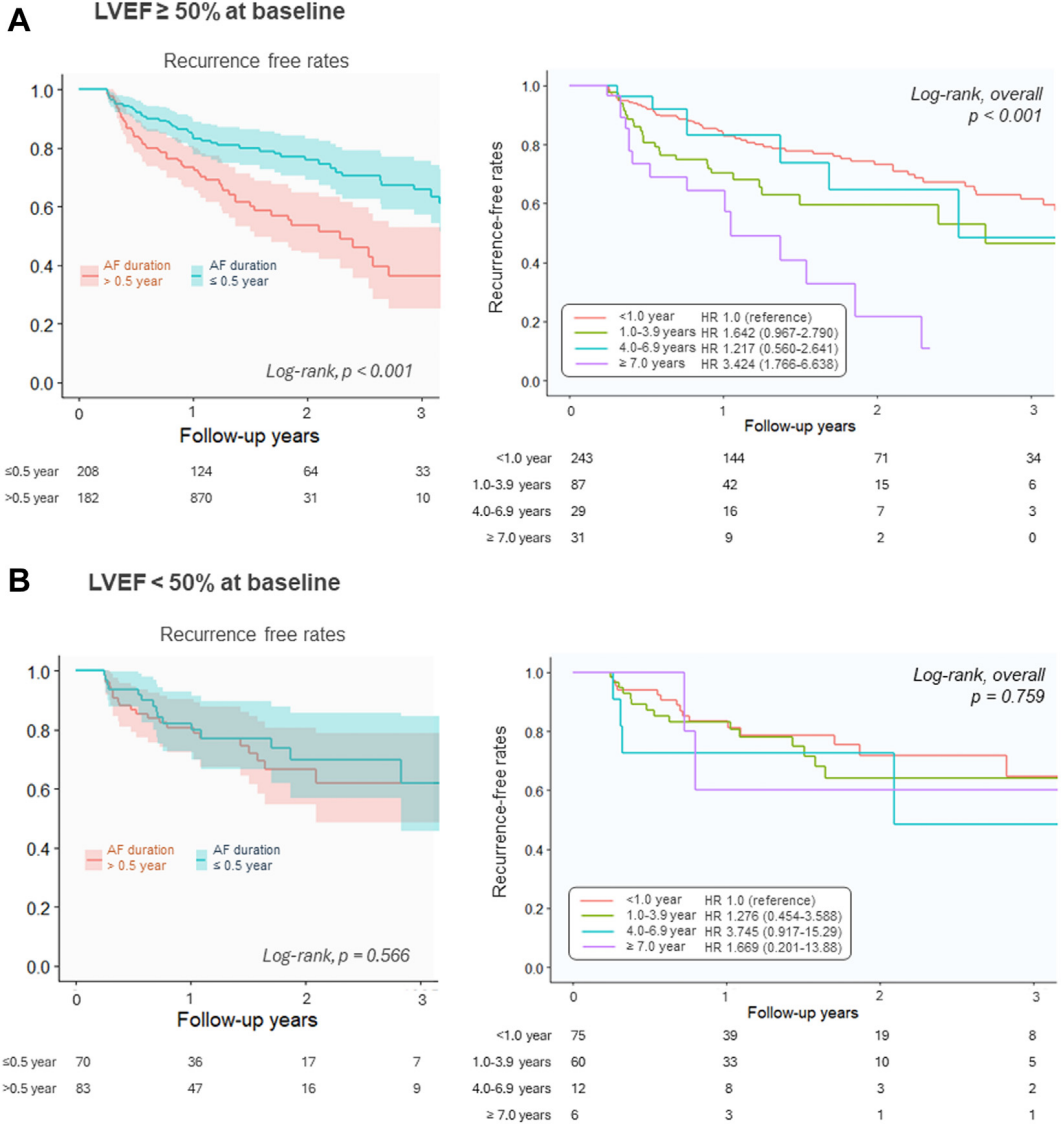
Recurrence in the Subgroups of Left Ventricular Ejection Fractions Kaplan-Meyer survival curves of recurrence after ablation between the early- and delayed-ablation groups among (A) patients with left ventricular ejection fraction (LVEF) ≥50% at baseline and (B) those with LVEF <50%. Comparison of the recurrence-free rates among 4 groups according to AF duration (<1.0 year, 1.0-3.9 years, 4.0-6.9 years, and ≥7.0 years) in each group are shown on the right, respectively. Abbreviation as in Figure 1.

**Figure 8:**
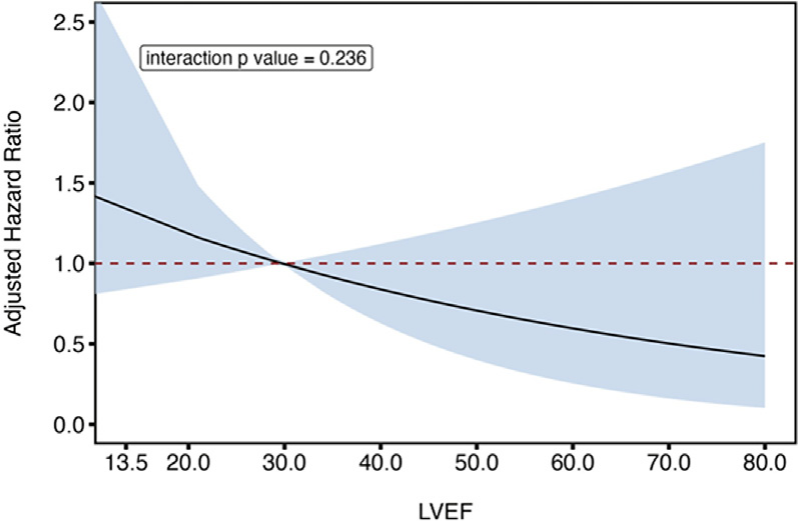
Recurrence Risk According to LVEF The effect of early ablation (≤0.5 year) vs delayed ablation (>0.5 year) on recurrence with LVEF function as a continuous variable. The figure shows the adjusted HR as a solid black line with the 95% CIs represented as the shaded area. Delayed ablation is used as the reference group. LVEF = left ventricular ejection fraction.

Among the patients with LVEF ≥ 50%, the recurrence-free rate was significantly higher in the early-ablation group than in the delayed-ablation group (75% [153/203] vs 63% [111/176]; *P* = 0.009) (Figure 7A). However, the difference in recurrence-free rates was not significant among patients with LVEF < 50% (76% [48/63] vs 73% [57/78]) in the early and delayed groups, respectively; *P* = 0.673) (Figure 7B). The recurrence-free rates differed significantly among the 4 groups divided by AF duration in patients with LVEF ≥50% (*P* < 0.001) but not in patients with LVEF <50% (*P* = 0.759) (Figures 7A and 7B). The HR for recurrence increased continuously along with decreased LVEF, with the largest estimated relative benefits for early ablation occurring in the preserved LVEF portion of the cohort (Figure 8). In contrast, no significant differences in all-cause death or HF hospitalization between the early- and delayed-ablation groups were observed in patients with LVEF ≥50% (log-rank test, *P* = 0.944 and *P* = 0.298, respectively) and those with LVEF <50% (log-rank test, *P* = 0.186 and *P* = 0.146, respectively).

### Risk of recurrence in the early- vs delayed-ablation groups across the subgroups stratified by baseline characteristics

**Figure 9:**
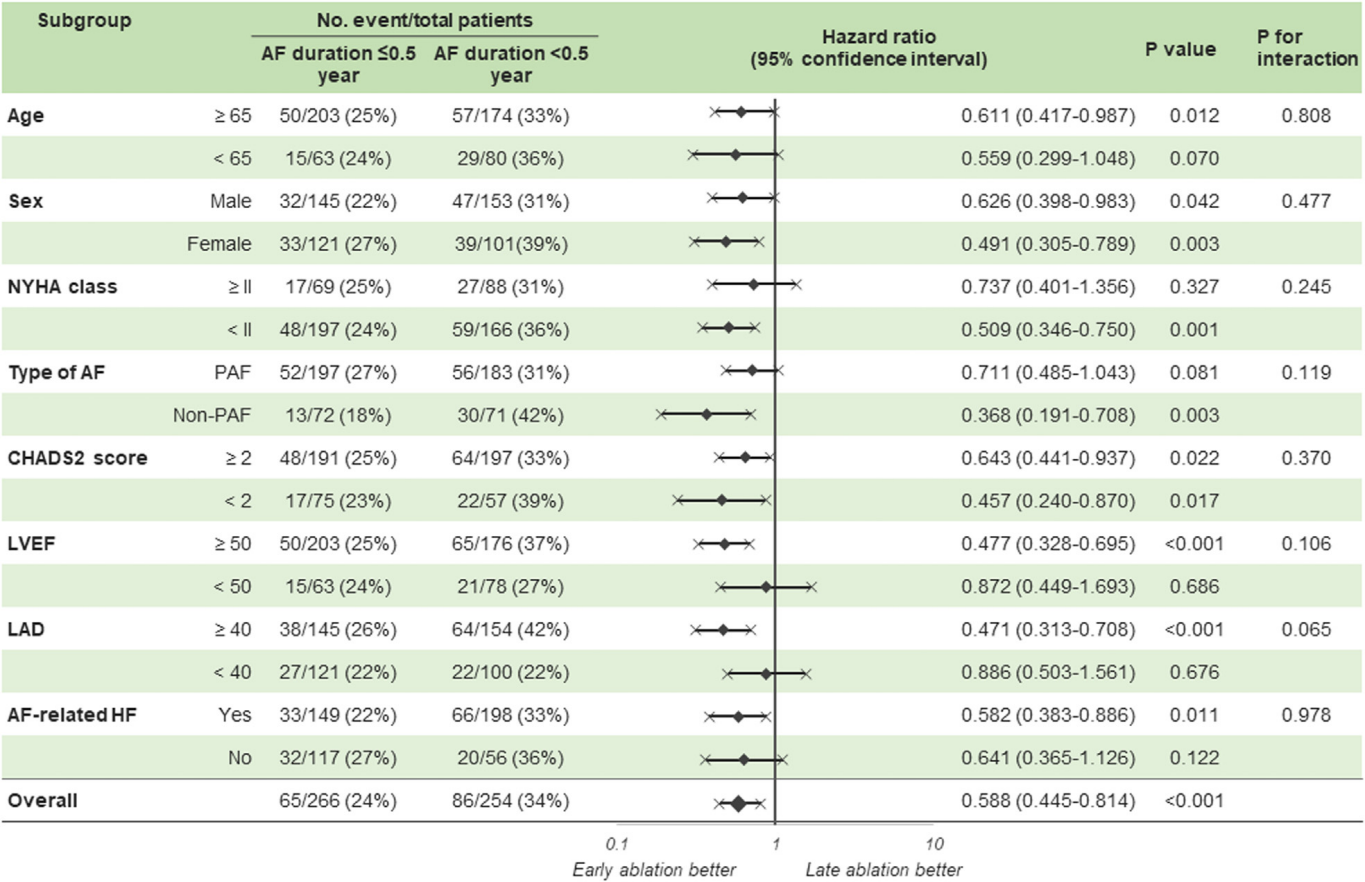
Recurrence Across the Subgroups Risk of recurrence for early-ablation vs delayed-ablation across the subgroups stratified by baseline characteristics. Abbreviations as in Figures 2 and 5.

Additional analyses to investigate which baseline characteristic category is related to the beneficial effect of early ablation for reducing recurrence are depicted in Figure 9. Although this beneficial effect was consistently observed across all subgroups, it was more likely to be relevant in patients with nonparoxysmal AF and LA diameter ≥40 mm in addition to the LVEF ≥50%.

## Discussion

The present study evaluated an effect of the duration between AF diagnosis and cryoballoon ablation on prognostic outcomes in patients with HF in a large-scale, multicenter study in Japan. Patient undergoing early cryoballoon ablation within 0.5 year after AF diagnosis experienced significantly less recurrence than those undergoing delayed ablation performed after 0.5 year. There was an incremental decrease in the recurrence rate that was associated with the decrease in AF duration. These results were predominantly observed in patients with preserved LVEF rather than those with systolic HF. Echocardiographic parameters and BNP levels improved after ablation regardless of the AF duration; however, the absolute values of chamber diameters were larger in the delayed-ablation group than in the early-ablation group at any time point. AAD use at baseline was independently associated with delayed ablation in patients with HF (Central Illustration).

**Central Illustration:**
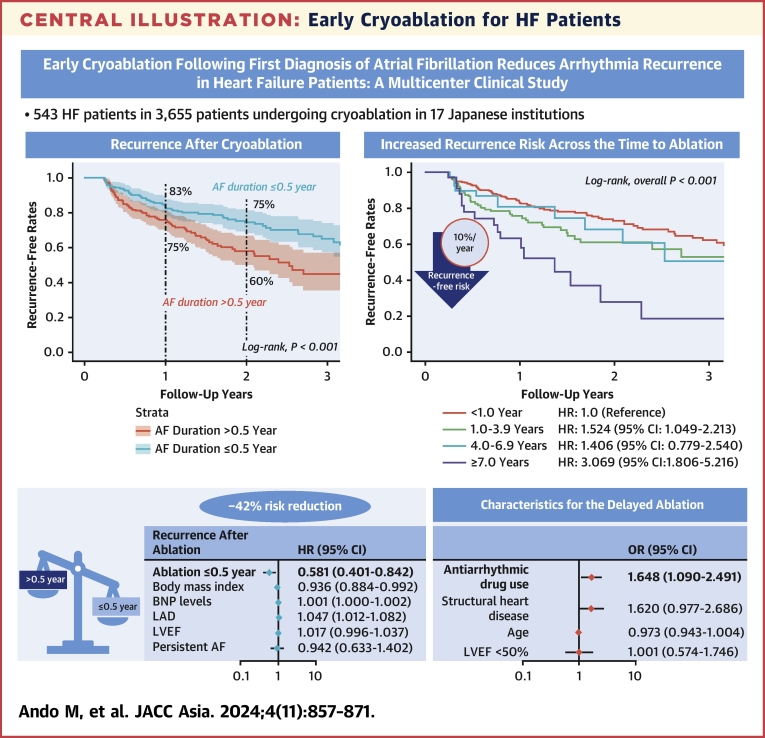
Early Cryoablation for HF Patients Early cryoablation from the first diagnosis of AF reduced the risk of recurrence in patients with HF. AF = atrial fibrillation; BNP = B-type natriuretic peptide; HF = heart failure; LAD = left atrial dimeter; LVEF = left ventricular ejection fraction.

For the last 2 decades, to the best of our knowledge, no studies have reported that catheter ablation improves the mortality of patients with AF; however, an improvement in recurrence rates superior to that of medical therapy has been reported.^10^ In contrast, the EAST-AFNET 4 trial showed an overall reduction in cardiovascular death and all-cause mortality with early rhythm control treatments.^3^ Notably, the rhythm control group in that study mostly used AADs, and their improved prognosis may have been driven from the enrolled patients’ characteristics with immediate rhythm control therapy after the diagnosis of AF within a median of 1 month.^3^ However, even first-line therapy with AADs may decrease in efficacy in cases of an extended period after the AF diagnosis. In the STOP AF study, the treatment success rate at 12 months was low at 45.0% in the AAD group, with a mean time from AF documentation to enrollment of 1.3 years.^11^ In contrast, the arrhythmia recurrence-free rate was greater in the ablation group (74.6%), although they had the same AF duration in that study.^11^ The favorable catheter ablation prognosis improved as the time between the first diagnosis of AF and ablation decreased, according to a previous report that used data from the institutional Healthcare Network of hospitals and clinics.^4^ Our findings were consistent with these reported results in demonstrating that early AF ablation was associated with decreased AF recurrence in patients with HF. The most prominent benefit of ablation was noted for patients who underwent the procedure <0.5 year after their AF diagnosis, whereas AAD use was independently associated with delayed ablation in patients with HF. Thus, first-line treatment using catheter ablation without AADs may suppress the recurrence of AF, which was also in line with the findings of a recent meta-analysis.^12^ In contrast, a recent randomized study failed to demonstrate the benefit of early ablation for AF on the recurrence-free rate compared with a 12-month delay in AF ablation.^13^ Nevertheless, the timing of catheter ablation in the course of management for the specific HF populations remains unclear.

HF is a complex and progressive disease.^14^ Acute exacerbations and rehospitalization over time can weaken or stiffen cardiac function, leading to debilitating physical and emotional symptoms. A dynamic process of atrial fibrotic remodeling, a possible cause of AF development, was progressively demonstrated in a magnetic resonance imaging study even in non-AF patients with HF over time.^15^ Once AF occurs in the presence of HF, it can worsen HF symptoms, resulting in a vicious cycle. Accordingly, catheter ablation for AF at an early stage can potentially break off this cycle. However, the early postdischarge period after HF hospitalization (vulnerable phase) carries a high risk of poor clinical outcome.^16^ This vulnerable phase persists 3 to 6 months after HF hospitalization discharge and may be explained by substantial subclinical hemodynamic abnormalities, residual neurohormonal and inflammatory activity, and insufficient dose adaptation of cardioprotective drugs.^16^ Although a risk is associated with administering an invasive intervention during this phase in patients with HF, the low invasiveness and high efficacy of cryoballoon ablation make it an ideal treatment for this population. Cryoballoon ablation offers myocardial necrosis with the possibility of suppressed inflammatory response in treating patients with AF.^17^ A previous study demonstrated that a short period from the diagnosis of AF to cryoablation for paroxysmal AF is positively associated with favorable outcomes.^18^ In our study, only 2 patients experienced worsening HF, and we demonstrated feasible procedural time results (approximately 130 minutes in total), reinforcing that cryoballoon ablation may be used as a first-choice treatment for this population. The reason for no significant difference in recurrence between the early- and delayed-ablation groups among patients with LVEF <50% is unclear; however, overall recurrence-free rates were not as low in both groups, implying that a mild-to-moderate systolic HF might be mostly involved in this population. Otherwise, it may be better to approach patients with HF at an early stage before the deterioration of the LVEF for a better recurrence prognosis, especially in AF-related HF. In addition, the results of the subgroup analysis may imply that early cryoablation may be more crucial in patients with progressive remodeling, such as large LA diameter and persistent AF.

Unfortunately, our study did not show significant differences in death and HF hospitalization rates between the groups. These results may be attributable to the few used endpoints and that most of the population consisted of patients with preserved LVEF. A recent observational study using the updated analyses of the institutional healthcare system demonstrated that delaying radiofrequency catheter ablation for AF results in increased all-cause mortality, HF hospitalization, and AF recurrence in patients with an LVEF >35% (approximately 20% of patients with HF).^19^ This result is supported by a recent large-scale study using a nationwide representative real-world cohort showing that catheter ablation for AF within 90 days after admission for HF was associated with improved cardiovascular outcomes and HF-related mortality.^8^ Notably, these studies only used radiofrequency catheter ablation because the use of cryoballoon ablation was not well established when the studies took place. The more recently developed approach of radiofrequency ablation and pulsed-field ablation in addition to cryoablation may further improve prognoses when used as an early intervention after the diagnosis of AF.

### Study limitations

This was a retrospective, nonrandomized study conducted in Japan. Baseline characteristics and examination results differed between the groups, and patients in the delayed-ablation group had a higher prevalence of comorbidities, consistent with a more-advanced atrial substrate at baseline. Moreover, uncountable bias and factors may have caused the clinicians to determine the timing of each cryoballoon ablation for their patients, requiring a randomized comparative study.^13^ A lead time bias was crucial in the current study because the delayed group comprised patients with advanced AF during the waiting time for ablation, with a relatively shorter mean time for recurrence after the procedure in the delayed group than that in the early group (362 days vs 446 days, *P* = 0.174). Because most patients had preserved LVEF and paroxysmal AF and the number of the patients with reduced LVEF was minimal, the study results were mostly driven from patients with AF-related HF. The ablation protocols and follow-up examinations to assess recurrence might have been different among the institutions, affecting the study’s findings. The rate of touch-up ablation for PV isolation was relatively high—nearly 30%—in our study. Because of the educational and training aspects of most institutions in the present study, clinical fellows and young doctors preferentially performed cryoballoon ablation because of its simplicity, which may be responsible for the decreased acute success rate and relatively high rate of major complications.^7^ The assessment for recurrence was mostly examined through short-term Holter monitoring (ie, 24-hour electrocardiogram monitoring), which was infrequently performed with variations among the institutions. Moreover, continuous monitoring such as implantable cardiac monitoring was not generally used, causing an underestimation of the recurrence rate. In addition, quality of life, patient physical activity level, and changes in AF-related symptoms after the ablation were not assessed in the present study. The follow-up times for assessing the BNP levels and echocardiography were not unified, and these values were not obtained from all patients. Finally, we defined the first diagnosis of AF as that given in an examination test clinically; however, subclinical, asymptomatic AF may have been present before the clinical detection, and this time discrepancy may have underestimated the AF duration in this study.

## Conclusions

The early timing of cryoballoon ablation from the first diagnosis of AF improved the recurrence-free rates in patients with HF. AAD administration was independently associated with a prolonged duration between AF diagnosis and the ablation. These findings may help improve the clinical management of these patients. Further well-organized, large-scale studies assessing the timing of cryoballoon ablation in patients with HF are warranted.

Perspectives **COMPETENCY IN MEDICAL KNOWLEDGE:** This large-scale, multicenter study demonstrated that AF recurrence rate was significantly lower in the early-ablation group than in the delayed-ablation group in patients complicated with HF, and early cryoballoon ablation incrementally reduced the risk of postablation recurrence. Our results imply the potential of an optimal timing of the treatment for HF patients with the better clinical management and prognosis.

**TRANSLATIONAL OUTLOOK:** This retrospective, nonrandomized study had some limitations. Baseline characteristics differed between the groups, and factors unaccounted for may have influenced clinicians in determining the timing of each ablation for their patients. Further prospective, randomized studies are required to assess the impact of the duration between the first AF diagnosis and cryoballoon ablation on the outcomes of HF patients.

## Funding Support and Author Disclosures

Drs Yanagisawa and Shibata are affiliated with a department sponsored by Medtronic Japan. All other authors have reported that they have no relationships relevant to the contents of this paper to disclose.
