# Supplementary material for: Early Cryoablation After First Diagnosis of Atrial Fibrillation Reduces Arrhythmia Recurrence in Heart Failure Patients
Source: JACC Asia. 2024 Sep 24;4(11):857–71. doi: 10.1016/j.jacasi.2024.08.005 (PMC11604533; doi:10.1016/j.jacasi.2024.08.005)
Supplement: Supplemental Table 1 and Supplemental Figures 1 and 2 [file mmc1.docx]

**Supplemental Table 1.** Institution information and number of the patients included in the study

| *Center* | *HF patients* | *Follow-up assessment after 12 months following ablation* |
| --- | --- | --- |
| Overall | 543 |  |
| Anjo Kosei Hospital | 32 | Outpatient care and ECG after 1, 3, 6, 9, and 12 months. A 24-h Holter monitoring after 6 and 12 months |
| Gifu Prefectural General Medical Center | 11 | Outpatient care and ECG after 1, 3, 6, and 12 months. A 24-h Holter monitoring after 6 months |
| Gifu Prefectural Tajimi Hospital | 9 | Outpatient care and ECG after 1, 3, 6, 9, and 12 months. A 24-h Holter monitoring after 3, 6, and 12 months |
| Gifu University Hospital | 6 | Outpatient care and ECG after 1, 3, 6, and 12 months. A 24-h Holter monitoring after 3 months |
| Japan Community Healthcare Organization Chukyo Hospital | 13 | Outpatient care and ECG after 2 weeks and 1, 3, and 6 months. A 24-h Holter monitoring after 3 months |
| Japanese Red Cross Aichi Medical Center Nagoya Daini Hospital | 288 | Outpatient care and ECG after 1, 3, 6, 9, and 12 months. A 24-h Holter monitoring after 3–4 and 12 months |
| Kainan Hospital | 3 | Outpatient care and ECG after ≤1, 3, 6, 9, and 12 months. A 24-h Holter monitoring after 3 and 6 months, and five-day Holter monitoring after 12 months |
| Kasugai Municipal Hospital | 6 | Outpatient care and ECG after ≤1, 3, 6, 9, and 12 months. A 24-h Holter monitoring after 1, 3 and 12 months |
| Komaki City Hospital | 12 | Outpatient care and ECG after 1, 3, 6, and 12 months. A 24-h Holter monitoring after 3, 6, and 12 months |
| Konan Kosei Hospital | 12 | Outpatient care and ECG after 1, 3, 6, 9, and 12 months. A 24-h Holter monitoring after 1, 6, and 12 months |
| Kuwana City Medical Center | 3 | Outpatient care and ECG every month. A 24-h Holter monitoring after 6 months |
| Nagoya City University East Medical Center | 10 | Outpatient care and ECG after 1, 3, 6, and 12 months. A 24-h Holter monitoring after 3 months |
| Nagoya City University Hospital | 11 | Outpatient care and ECG after 1, 3, 6, and 12 months. A 24-h Holter monitoring after 3, 6, and 12 months |
| Nagoya University Hospital | 30 | Outpatient care and ECG after 1, 3, 6, 9, and 12 months. A 24-h Holter monitoring after 1 month |
| Ogaki Municipal Hospital | 63 | Outpatient care and ECG after 1, 3, 6, and 12 months. A 24-h Holter monitoring after 1, 3, 6, and 12 months |
| Toyota Memorial Hospital | 18 | Outpatient care and ECG after 1, 3, 6, and 12 months. A 24-h Holter monitoring after 3, 6, and 12 months |
| Yokkaichi Municipal Hospital | 13 | Outpatient care and ECG after 1, 3, 6, and 12 months. A 24-h Holter monitoring after 3, 6, and 12 months |

ECG, electrocardiography; HF, heart failure

**Supplemental Figure 1**


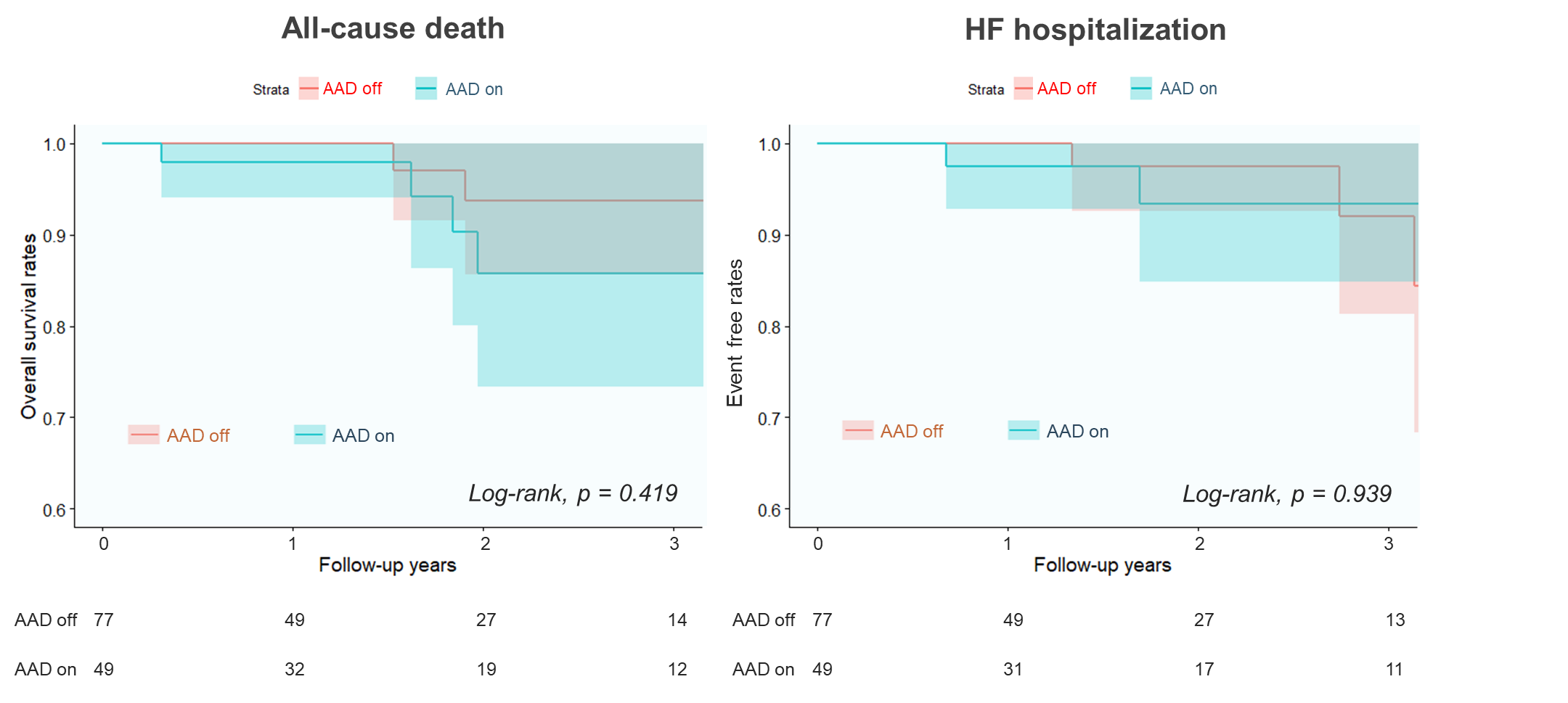


Comparisons of mortality (A) and HF hospitalization (B) between patients who continued AADs and those who did not post-ablation in patients administered AADs preoperatively and maintained sinus rhythm after ablation. AAD, anti-arrhythmic drugs; HF, heart failure.

**Supplemental Figure 2**


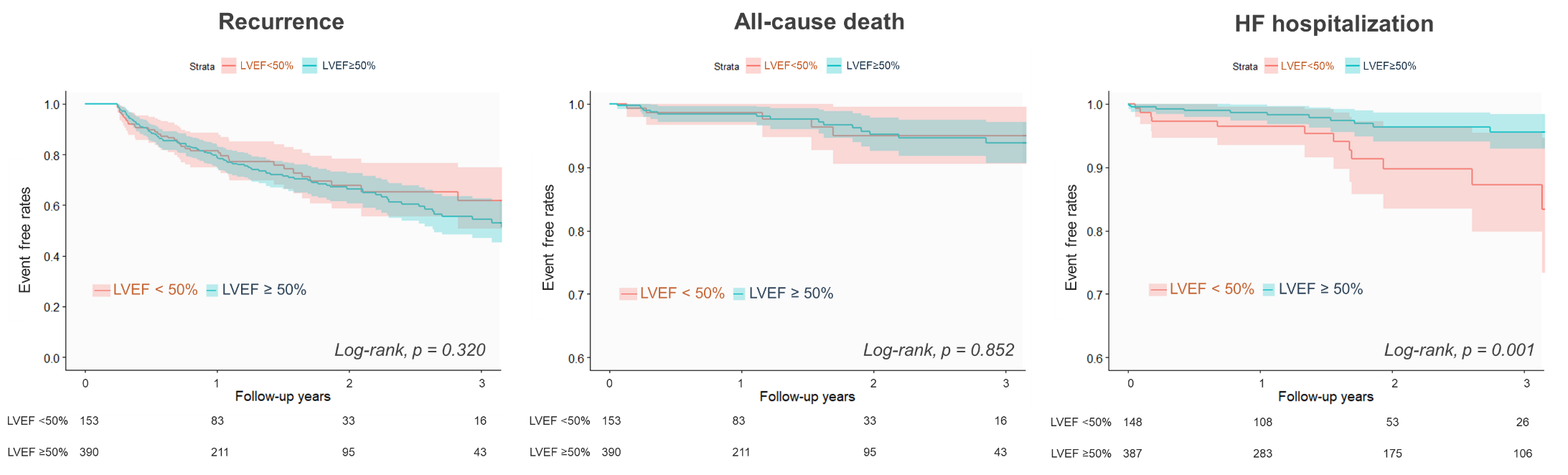


Comparisons of recurrence (A), mortality (B), and HF hospitalization (C) between patients with LVEF <50% and those with LVEF ≥50% after ablation. HF, heart failure; LVEF, left ventricular ejection fraction.
